# Cutaneous Manifestations in Patients with Chronic Kidney Disease on Maintenance Hemodialysis

**DOI:** 10.5402/2012/679619

**Published:** 2012-07-05

**Authors:** Praveen Kumar Kolla, Madhav Desai, Ram Mohan Pathapati, B. Mastan Valli, Suneetha Pentyala, G. Madhusudhan Reddy, A. Vijaya Mohan Rao

**Affiliations:** ^1^Department of Nephrology, Narayana Medical College Hospital, Chinthareddypalem, Nellore 524002, India; ^2^Department of Clinical Pharmacology, Narayana Medical College Hospital, Chinthareddypalem, Nellore 524002, India; ^3^Department of Radiology, Narayana Medical College Hospital, Chinthareddypalem, Nellore 524002, India; ^4^Department of Dermatology, Narayana Medical College Hospital, Chinthareddypalem, Nellore 524002, India

## Abstract

Cutaneous disorders can precede or follow the initiation of hemodialysis treatment. We evaluated the prevalence of various dermatological manifestations in patients undergoing hemodialysis at least twice a week for minimum of three months at our center. Patients were excluded if they were undergoing hemodialysis less than twice a week or on hemodialysis secondary to ESRD following graft dysfunction. One hundred and forty-three patients were evaluated. Among them, there were 113 male and 30 females. Among the skin changes, pruritus accounted for 56%, Xerosis was observed in 52%, Diffuse blackish hyper pigmentation was seen in 40%. Skin infections was seen in 53% of patients, of these fungal, bacterial and viral infections were 27.2%, 14.6%, and 11.2%, respectively. Kyrle's disease was observed only in 6.9%. Other skin manifestations include eczema 4.8%, psoriasis 2.7%, and drug rash 2.1%. Nail changes were observed in 46 patients of whom 27 patients had onychomycosis. Other changes include discoloration, onycholysis, and splinter hemorrhages. Hair changes were observed in 21.7%. Mucosal changes were seen in 27.3%. In our study, pruritus, xerosis, and pigmentation were higher among skin changes. Recognition and management of some of these dermatological manifestations vastly reduce the morbidity and improve the quality of life.

## 1. Introduction

Chronic kidney disease (CKD) is a progressive loss of kidney function over a period of months or years through five stages. The number of patients with end-stage renal disease (ESRD) in India is increasing with an estimated annual incidence of about 100 per million populations [1]. Hemodialysis is one of the therapeutic modalities which can improve the survival in these patients [2]. About 50–100% of patients with ESRD have at least one associated cutaneous change [3, 4]. These cutaneous disorders can precede or follow the initiation of hemodialysis treatment, and there are more chances to develop newer skin changes during the course of hemodialysis therapy, which may affect the quality of life. We evaluated the prevalence of various dermatological manifestations in patients undergoing hemodialysis at our center.

## 2. Materials and Methods

Patients undergoing regular hemodialysis at least twice a week for a minimum of three months were evaluated. Patients were excluded if they were undergoing hemodialysis less than twice a week or on hemodialysis secondary to ESRD following graft dysfunction. All patients underwent clinical examination, and relevant investigations were recorded. Dermatological evaluation and confirmation of the presenting lesions were done by qualified dermatologist. Microscopic evaluation for fungal infections and histopathologic evaluation for Kyrle disease was performed.

## 3. Statistical Analysis 

All the data was entered in Microsoft Excel spread sheet. Statistical analysis was done by using Graphpad Prism, version 4, USA. Continuous data was described as arithmetic mean and standard deviation and categorical data as actual numbers and percentages. 

## 4. Results

One hundred and forty-three patients undergoing regular hemodialysis twice a week were evaluated. Among them, there were 113 male and 30 females (3.67 : 1). The mean age of these patients was 43.8 ± 13.0 yrs (14–70). The mean values of serum creatinine, blood urea, hemoglobin, uric acid, and kt/v of these patients were 9.4 ± 6.3 mg/dL, 133.5 ± 46.9 mg/dL, 7.17 ± 1.7 gm, 7.0 ± 1.8 mg/dL, and 1.2 ± 0.1, respectively. The mean duration of dialysis was 23.0 ± 14.1 months. The causes of ESRD and cutaneous manifestations were described in Table 1. 

**Figure 1:**
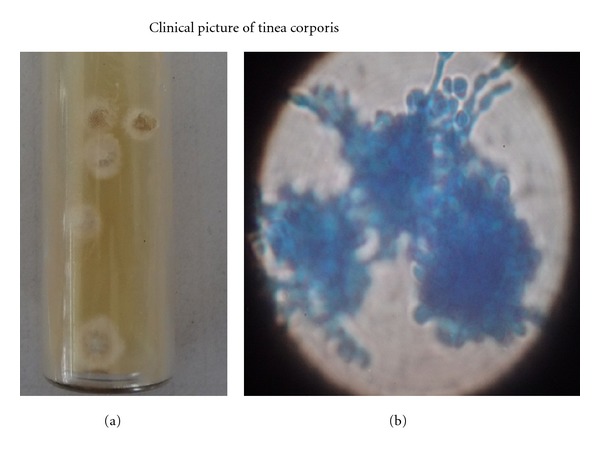
Microscopy of tenia corporis.

**Figure 2:**
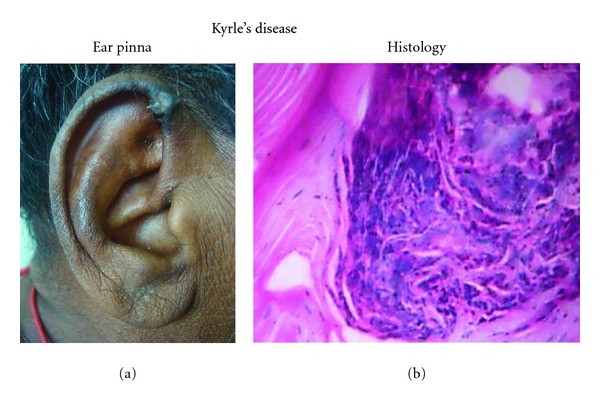
Kyrle's disease and histopathology.

Among the skin changes, pruritus accounted for 56% of the total study population. It manifested even before hemodialysis in 28 patients, and only 4 patients out of 81 showed significant improvement after hemodialysis. Pruritus was found to be severe in diabetic patients (21/37). Xerosis was observed in 52% of the study population. Diffuse blackish hyperpigmentation was seen in 40% and was predominant in sun-exposed areas. 53% of patients had skin infections, of these fungal, bacterial and viral infections were 27.2%, 14.6%, and 11.2%, respectively. Among the fungal infections onychomycosis, tinea versicolor and tinea cruris (Figure 1) were the most common, and among the viral infection herpes labialis and herpes zoster were common. Kyrle's disease (Figure 2) was observed only in 6.9% in our patients. Other skin manifestations include eczema 4.8%, psoriasis 2.7%, and drug rash 2.1%. 

**Figure 3:**
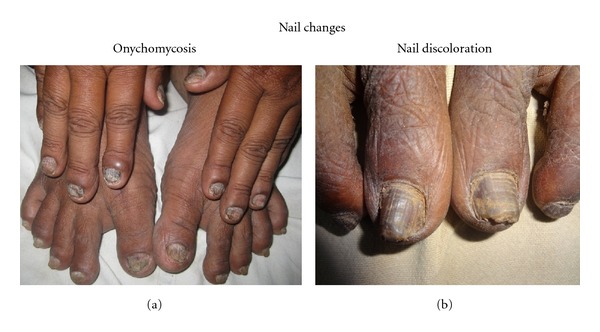
Nail changes of patients undergoing hemodialysis.

Nail changes were observed in 46 patients of whom 27 patients had onychomycosis (Figure 3). Other changes include discoloration, onycholysis, and splinter hemorrhages. Hair changes were observed in 21.7%. Mucosal changes were seen in 27.3%. 

## 5. Discussion

Pruritus was the most common skin manifestation observed in our patients as compared to other studies [5]. Pruritus was found to be severe in diabetics. Dry luster less skin could have contributed for such a high percentage in our study group. Xerosis was found to be the second most common manifestation which predominantly affected extensor surfaces of forearms, legs, and thighs and was severe in diabetics [6]. Similar observation was noticed in previous studies with a prevalence of 45–90% [7]. High dosage of diuretics, reduction in size of sweat glands, and excessive ultrafiltration might be responsible for the above manifestation. Emollients were prescribed to these patients and found to be effective.

Diffuse blackish hyperpigmentation was seen in 40%, which was relatively high when compared to 20–22% in other studies [8]. This might be probably due to the failure of kidneys to excrete beta-melanocyte stimulating hormone and resultant melanin being deposited in basal layer as well as superficial dermis. Skin infections were comparatively lesser in proportion when compared to Udayakumar et al. [5] and Bencini et al. [9] observations. The high incidence of infections might be due to diabetes, low albumin, elevated intracellular calcium, acidosis, or repetitive vascular procedures. Among the acquired perforating disorders, only Kyrle's disease was noticed, and others like perforating folliculitis, and perforating collagenosis were not found in our study. 

Nail changes observed in our study were onychomycosis, discolouration, onycholysis, and splinter hemorrhages. Hair changes were observed with a sparse distribution over body which included dryness and hair discoloration. Dryness was possibly due to decreased sebum secretion. This was high compared to 10–30% in other studies [10, 11]. The reported incidence of oral mucosal changes in hemodialysis patients was 27.3% compared to 90% in previous studies [12]. Changes observed in our study were xerostomia, angular cheilitis, gingivitis, and uremic breath. Possible causes include dehydration, mouth breathing, and high concentration of urea, and failure to breakdown into ammonia.

## 6. Conclusions

In our study, pruritus, xerosis, and pigmentation were higher among skin changes. Recognition and management of some of these dermatological manifestations vastly reduce the morbidity and improve the quality of life.

## Figures and Tables

**Table 1:** The causes of ESRD and cutaneous manifestations in patients undergoing hemodialysis.

Dermatological manifestations	Total	HTN	DM	CGN	CIN	Others
	*N* = 143 (100%)	*N* = 78 (52.4%)	*N* = 37 (25.9%)	*N* = 15 (10.5%)	*N* = 7 (4.9%)	*N* = 9 (6.3%)
Skin changes						
Pruritus	81 (56.6)	43	21	9	2	6
Xerosis	74 (51.7)	38	22	4	4	6
Pigmentation	56 (39.4)	15	26	9	2	2
Fungal	39 (27.2)	15	17	4	1	2
Bacterial	21 (14.6)	15	4	1	1	0
Viral	17 (11.2)	11	3	2	1	1
Acne	14 (9.7)	6	4	2	2	0
Kyrles	10 (6.9)	3	5	1	0	1
Eczema	7 (4.8)	4	1	2	0	0
Psoriasis	4 (2.7)	3	1	0	0	0
Drug rash	3 (2.1)	2	0	0	0	1

Nail Changes						
Discoloration	13 (9.09)	10	3	0	0	0
Onychomycosis	27 (18.88)	10	11	5	0	1
Onycholysis	3 (2.10)	2	1	0	0	0

Hair and mucosal changes						
Splinter hemorrhages	3 (2.10)	0	1	1	1	0
Hair changes	31 (21.7)	17	11	2	1	0
Mucosa	39 (27.3)	20	5	5	5	4

HTN: hypertension, DM: diabetes mellitus, CGN: chronic glomerular nephritis, CIN: chronic interstitial nephritis.
